# Association of type 2 diabetes remission and risk of cardiovascular disease in pre‐defined subgroups

**DOI:** 10.1002/edm2.280

**Published:** 2021-06-19

**Authors:** Hilda Hounkpatin, Beth Stuart, Andrew Farmer, Hajira Dambha‐Miller

**Affiliations:** ^1^ School of Primary Care Population Sciences and Medical Education, University of Southampton Southampton UK; ^2^ Primary Care Research Centre University of Southampton Southampton UK; ^3^ Nuffield Department of Primary Care University of Oxford Oxford UK

**Keywords:** epidemiology, healthcare delivery, lifestyle, macrovascular disease, microvascular disease

## Abstract

**Aim:**

To quantify the association between type 2 diabetes remission and 5‐year incidence of cardiovascular disease outcomes, overall and in pre‐defined subgroups.

**Methods:**

Retrospective cohort analysis of 60,287 adults with type 2 diabetes from the Care and Health Information Analytics (CHIA) database. Multivariable Cox models were used to assess the association between remission within the first two years of follow‐up and incidence of cardiovascular disease (CVD) outcomes including events, microvascular and macrovascular complications at 7‐year follow‐up. Effect modification by age, sex, diabetes duration, pre‐existing CVD, baseline body mass index (BMI) and HbA_1c_ level was assessed.

**Results:**

7489 (12.4%) people achieved remission during the first two years of follow‐up. Overall, remission was associated with lower risk of CVD outcomes. Remission was associated with lower risk of microvascular complications for younger compared with older age groups (eg aHR: 0.59 (0.41–0.84) and aHR: 0.78 (0.67–0.92) for those aged <45 years and 75–84 years, respectively). Amongst those achieving remission, those with no or 1–2 comorbidities had lower risk of microvascular complications (aHR: 0.65 (0.56–0.75)) compared to those with more than three comorbidities (aHR: 0.83 (0.69–0.99), respectively). There were no significant interactions in the remaining subgroups or for models assessing CVD events or macrovascular complications.

**Conclusions:**

Achieving remission of type 2 diabetes is associated with a lower risk of microvascular complications, particularly for younger groups and those with fewer comorbidities. Targeted interventions that focus on promoting remission in these groups may reduce the impact of microvascular complications and associated health costs.

## Abstract 2

Remission of type 2 diabetes has been shown to be achievable through lifestyle changes alone. The extent to which remission is associated with reduced cardiovascular disease (CVD) outcomes in key subgroups of people with type 2 diabetes has not previously been assessed. We found that achieving remission of type 2 diabetes was associated with a lower risk of microvascular complications, particularly for younger groups and those with no or few comorbidities. Targeted interventions that focus on promoting remission in these groups may reduce the impact of microvascular complications and associated health costs.

## 1 INTRODUCTION

Type 2 diabetes is a progressive chronic condition affecting over 460 million adults globally and is associated with significantly increased risk of cardiovascular disease (CVD) and mortality.^1^, ^2^ CVD costs the National Health Service (NHS) approximately £9 billion a year, with much higher costs to wider UK economy.^3^ A growing body of evidence from clinical trials has shown that biochemical remission of diabetes, defined as a level of glycaemia below a diagnostic threshold (HbA_1c_ < 6.5% or 48 mmol/mol) in the absence of pharmacological or surgical intervention, is achievable through lifestyle management.^4^, ^5^, ^6^, ^7^, ^8^, ^9^ In the Look AHEAD study, participants who achieved weight loss during the intensive lifestyle intervention experienced reduced cardiovascular events over a median follow‐up of 9.6 years.^10^ However, 15% of participants with well‐controlled diabetes and poor self‐reported general health experienced increased risk of CVD events and CVD‐related mortality. Averaged across 4 years, intensive lifestyle intervention participants had a greater percentage of weight loss than diabetes support and education participants; however, in participants without CVD at baseline, CVD events were reduced by 14% (274 vs 240; 1.42 vs 1.23%) while those with CVD (14%) experienced an increase in CVD events of 13% (144 vs 163; 5.92 vs 6.56%), although this difference was statistically insignificant.^11^ Recently, the Association of British Clinical Diabetologists (ABCD) and the Primary Care Diabetes Society (PCDS) have acknowledged that any weight loss—including unintentional weight loss—may contribute to remission.^12^ It is likely that remission may be associated with reduced risk of cardiovascular events for some people; however, for others (eg those with pre‐existing CVD or cancers) weight loss and remission may be harmful and associated with adverse events.

There is currently a lack of studies exploring whether the association between remission and CVD risk varies by certain subgroups. It could be valuable to understand determinants that predict CVD events in people who achieve remission to allow interventions to be targeted and personalised. In the present study, we therefore sought to examine the remission‐CVD link overall and in pre‐defined subgroups using a large population‐based cohort with established type 2 diabetes from routine clinical care.

## 2 PARTICIPANTS AND METHODS

### 2.1 Study population

We used data from the Care and Health Information Analytics database (CHIA), a pseudo‐anonymised live electronic database linking routinely collected primary care data for approximately 1.5 million people from 150 general practitioner (GP) practices across Hampshire and Isle of Wight (Southern England, UK) with clinical biochemistry data from local hospital laboratories. We identified a cohort of 60,715 adults (aged 18–85 years) over 7 years who were coded for type 2 diabetes based on the Quality and Outcomes Framework (QOF) Read code criteria prior to 1 January 2013, and who had continuously recorded electronic records over seven years from the 1 January 2013 to 1 April 2020.

### 2.2 Exposure definition

Remission (at any point during the first two years of the study period: 1 January 2013–2031 December 2015) was defined as two consecutive HbA_1c_ level <48 mmol/mol (6.5%) measurements separated by a minimum period of 6 months in the absence of diabetes medications (assessed in 6‐month intervals) or bariatric surgery.^9^

### 2.3 Baseline characteristics

Baseline data on sociodemographic characteristics such as age, sex and ethnicity (White, Black, Asian, Mixed and other), socio‐economic status (defined using the 2019 Index of Multiple Deprivation (IMD) quintiles, a small‐area measure of socio‐economic status, ranked nationally and comprises seven domains: income, employment, education/skills/training, health and disability, crime, barriers to housing and services, and living environment) were available. Clinical variables at baseline included the following: comorbidities (defined from diagnostic codes from existing QOF conditions—coronary heart disease, chronic kidney disease, chronic obstructive pulmonary disease (COPD), asthma, cancer, dementia, atrial fibrillation, epilepsy, heart failure, stroke, peripheral vascular disease, hypertension, osteoporosis, osteoarthritis and depression), frailty (defined using the electronic frailty index score), latest smoking status, weight and biochemistry measures (eg glycated haemoglobin (HbA_1c_), total cholesterol, HDL‐cholesterol, eGFR).

### 2.4 Outcomes

The outcomes of interest were CVD outcomes including events (defined as a composite of myocardial infarction (MI), amputation and stroke), microvascular complications (comprising peripheral neuropathy, retinopathy and nephropathy) and macrovascular complications (comprising stroke, MI, coronary heart disease (CHD), peripheral arterial disease (PAD) or amputation) occurring between years 2 and 7 of follow‐up. QoF definitions for each diagnosis were used.

### 2.5 Statistical analysis

Missing data on biochemistry variables, remission status, baseline weight and IMD were assumed to be missing at random and multiply imputed using chained equations using STATA SE 16.0. Ten cycles of imputation were used. All imputed data after patient death were recoded as missing. Missing data on ethnicity were recoded as white ethnicity.

Descriptive statistics were used to compare baseline sociodemographic and clinical characteristics for individuals who did and those who did not achieve remission during the first 2 years of follow‐up. Cox proportional hazards models were fitted to estimate the association between remission in the first 2 years of follow‐up and 5‐year incidence of CVD events, macrovascular complications and microvascular complications. These models included only those alive at the end of the first 2 years of follow‐up. The mid‐point of the quarter of death was used as exact date of death was not available. Multivariable models were adjusted based on a priori reasoning for age, sex, ethnicity, IMD, pre‐existing CVD, BMI, diabetes duration, number of comorbidities and clustering within practices (all at baseline). We additionally adjusted for last observed BMI and hypertension status. We then modelled interaction terms between remission and age (<45, 45–54,55–64, 75–84, 85+), sex (male, female), diabetes duration (<5, 5–<10, 10–<20, 20+ years), pre‐existing CVD (no/yes; defined as a composite of myocardial infarction (MI), amputation, and stroke), baseline BMI [underweight (<18.5kg/m^2^), normal (18.5–24.9kg/m^2^), overweight (25–29.9kg/m^2^), obese (≥30kg/m^2^)],^13^ baseline HbA_1c_ [<6.5%) (48mmol/mol); 6.5–8% (48–63.9 mmol/mol); 8–9% (64–74.9 mmol/mol); >9% (75mmol/mol)] ^14^ and number of comorbidities (0, 1–2, 3+) in models additionally adjusting for ethnicity and IMD. Cox regression models stratified by subgroups identified as statistically different were fitted. Analyses were repeated after excluding those with pre‐existing CVD, microvascular and macrovascular complications at baseline.

## 3 RESULTS

### 3.1 Study cohort characteristics

The study cohort included 60,287 people with type 2 diabetes for whom remission status could be assessed (ie had HbA_1c_ data at baseline and at least one follow‐up measurement). The cohort was followed for a mean of 6.8 (SD = 1.3) years. Table 1 summarises baseline characteristics of the cohort. The mean age of the cohort was 64.6 (12.0) years, and mean duration diabetes was 8.1 (6.8) years. Most were male: 34,408 (57.1%) and of white ethnicity: 58,148 (96.5%). A significant proportion had microvascular (18,678 (31.0%)) or macrovascular (113,453 (18.8%)) complications at baseline. During the first 2 years of follow‐up, 7489 (12.4%) people achieved remission. Those achieving remission were more likely to be older, female, non‐smokers, living in less deprived areas and with a shorter duration of diabetes (Table 1).

**TABLE 1:** Baseline characteristics of the CHIA type 2 diabetes remission cohort^a^

	All (*n =* 60,287)	No remission (*n* = 52,798)	Remission (*n* = 7489)	*p*‐Value
Sociodemographic				
Age, y^b^	64.6 (12.0)	64.2 (12.0)^c^	67.0 (11.4)	<.001
Male gender, *n* (%)	34,408 (57.1)	30,453 (57.7)	3956 (52.8)	<.001
Ethnicity, *n* (%)				
White	58,148 (96.5)	50,839 (96.2)	7309 (97.6)	
Black	217 (0.4)	198 (0.4)	19 (0.3)	.193
Asian	1514 (2.5)	1395 (2.7)	119 (1.6)	.001
Mixed/Other	408 (0.7)	366 (0.7)	41 (0.6)	.189
Socio‐economic status, *n* (%)				
Index of multiple deprivation quintile 1 (most deprived)	7576 (12.6)	6858 (13.0)	718 (9.6)	<.001
Index of multiple deprivation quintile 2	12,137 (20.1)	10,695 (20.3)	1443 (19.3)	
Index of multiple deprivation quintile 3	11,457 (19.0)	10,180 (19.3)	1276 (17.0)	
Index of multiple deprivation quintile 4	13,028 (21.6)	11,298 (21.4)	1729 (23.1)	
Index of multiple deprivation quintile 5 (least deprived)	16,090 (26.7)	13,767 (26.1)	2322 (31.0)	
Clinical				
Diabetes duration, years (*n* = 60,138)	8.1 (6.8)	8.5 (7.0)	5.9 (5.2)	<.001
Frailty index (*n* = 60,244)	0.2 (0.1)	0.2 (0.1)	0.2 (0.1)	.173
Total number baseline comorbidities, *n*(%)	1.3 (1.2)	1.3 (1.2)	1.4 (1.2)	<.001
Hypertension, *n* (%)	30,868 (51.2)	26,851 (50.9)	4017 (53.6)	.001
Stroke, *n* (%)	2584 (4.3)	2245 (4.3)	339 (4.5)	.449
Myocardial infarction, *n* (%)	4208 (7.0)	3763 (7.1)	446 (5.9)	.005
Amputation, *n* (%)	648 (1.1)	600 (1.1)	48 (0.6)	.003
Current smoker, *n* (%)	6559 (10.9)	5838 (11.1)	721 (9.6)	.003
Microvascular complications	18,678 (31.0)	17,210 (32.6)	1468 (19.6)	<.001
Macrovascular complications	11,345 (18.8)	9990 (18.9)	1355 (18.1)	<.001
Weight, kg^a^	90.9 (20.7)	91.3 (20.7)	88.0 (20.1)	<.001
BMI, kg/m^2a^	31.5 (6.3)	31.7 (6.3)	30.6 (6.1)	<.001
Systolic blood pressure, mmHg^a^	136.1 (15.4)	136.1 (15.4)	136.0 (15.4)	.623
Diastolic blood pressure, mmHg^a^	77.2 (9.4)	77.3 (9.4)	76.9 (9.3)	.020
Total cholesterol, mmol/L^a^	4.6 (1.2)	4.5 (1.2)	4.7 (1.2)	<.001
HDL‐cholesterol, mmol/L^a^	1.2 (0.4)	1.2 (0.3)	1.3 (0.4)	<.001
HbA_1c_ level, mmol/mol^a^	60.1 (20.4)	60.2 (20.5)	59.1 (19.5)	.331
eGFR	73.1(17.1)	73.2 (17.1)	72.5 (17.0)	.010
Total number of medications prescribed^d^	3.9 (2.4)	4.0 (2.4)	3.3 (2.3)	<.001
Anti‐hypertensive medication, *n* (%)	32,509 (53.9)	28,386 (53.8)	4123 (55.1)	.115
Lipid‐lowering medication, *n* (%)	40,992 (68.0)	36,344 (68.8)	4648 (62.1)	<.001
Hypoglycaemic medication, *n*(%)	41,085 (68.1)	38,764 (73.4)	2320 (31.0)	<.001

^a^ Remission assessed for those with HbA_1c_ measurement at baseline and at least one HbA_1c_ measurement in the first two years of the follow‐up period, ie year 0–2(*n* = 60,287). Remission was defined as having two consecutive HbA1c < 6.5% (48 mmol/mol) measurements separated by a minimum period of 6 months and no oral hypoglycaemics and no history of bariatric surgery.

^b^ Mean (SD).

^c^ Estimation sample varies across imputations; minimum number of observations reported.

^d^ Medication was defined as being prescribed during the first 6 months of the follow‐up year (ie January‐July 2013). Microvascular complications included a composite of peripheral neuropathy, retinopathy and nephropathy. Macrovascular complications include a composite of stroke, MI, coronary heart disease, peripheral arterial disease (PAD) and amputation.

### 3.2 Remission and CVD outcomes

Three thousand four hundred and eighteen (5.7%), 12121 (20.1%) and 4839 (8.0%) people had a CVD event, microvascular or macrovascular complication during the last 5 years of follow‐up. Compared with those who did not, those who achieved remission had a significantly lower risk of CVD (aHR: 0.75 (0.64–0.88)), microvascular complications (aHR: 0.68(0.62–0.74)) or macrovascular complications (aHR: 0.78 (0.69–0.89)) at 7 years follow‐up. Univariate‐ and multivariate‐adjusted associations are presented in Table 2. Similar results were observed amongst those who did not have pre‐existing CVD, microvascular or macrovascular complications at baseline.

**TABLE 2:** Association between remission and incidence of CVD outcomes over five‐year follow‐up in the CHIA type 2 diabetes cohort

	CVD event			Microvascular complications			Macrovascular complications		
	*N*	HR (95% CI)	*p*‐Value	*N*	HR (95% CI)	*p*‐Value	*N*	HR (95% CI)	*p*‐Value
**Unadjusted**	59,628			59,627			59,628		
Remission									
No		1			1			1	
Yes		0.74 (0.64–0.87)	<.001		0.68 (0.63–0.74)	<.001		0.78 (0.69–0.88)	<.001
Maximally adjusted^a^	59,483			59,482			59,483		
Remission									
No		1			1			1	
Yes		0.75 (0.64–0.88)	.001		0.68 (0.62–0.74)	<.001		0.78 (0.69–0.89)	<.001
Baseline weight		1.00 (0.99–1.00)	.011		1.00 (1.00–1.00)	.882		0.99 (0.99–1.00)	<.001
Diabetes duration		1.02 (1.02–1.03)	<.001		0.99 (0.98–0.99)	<.001		1.02 (1.02–1.02)	<.001
Pre‐existing CVD		0.61 (0.55–0.69)	<.001		1.02 (0.96–1.07)	.599		0.67 (0.61–0.74)	<.001
Total number comorbidities		1.09 (1.06–1.12)	<.001		0.98 (0.96–1.00)	.039		1.08 (1.05–1.11)	<.001
BMI		0.99 (0.99 – 1.00)	.062		1.00 (1.00–1.00)	.497		1.00 (0.99–1.00)	.114
Hypertension		0.86 (0.79–0.93)	<.001		1.03 (0.98–1.08)	.313		1.18 (1.09–1.27)	<.001
Age		1.02 (1.02–1.02)	<.001		0.99 (0.99–0.99)	<.001		1.02 (1.01–1.02)	<.001
Gender		1.47 (1.35–1.60)	<.001		1.00 (0.96–1.05)	.938		1.51 (1.41–1.61)	<.001
Ethnicity									
White		1			1			1	
Black		0.62 (0.30–1.26)	.187		1.22 (0.95–1.56)	.121		0.80 (0.46–1.37)	.412
Asian		0.65 (0.51–0.83)	<.001		1.57 (1.18–2.08)	.002		0.71 (0.59–0.87)	.001
Mixed/Other		1.13 (0.77–1.66)	.535		1.42 (1.15–1.79)	.001		0.95 (0.65–1.40)	.796
IMD									
Q1 (Most deprived)		1			1			1	
Q2		0.85 (0.75–0.96)	.007		1.13 (1.00–1.29)	.051		0.85 (0.75–0.96)	.007
Q3		0.81 (0.71–0.92)	.001		1.02 (0.91–1.14)	.695		0.82 (0.73–0.93)	.002
Q4		0.82 (0.72–0.93)	.002		1.06 (0.94–1.20)	.346		0.85 (0.75–0.96)	.010
Q5 (Least deprived)		0.76 (0.67–0.87)	<.001		1.14 (0.99–1.31)	.079		0.80 (0.71–0.91)	.001

^a^ Maximally adjusted models adjusted for baseline weight, sociodemographic variables, diabetes duration, pre‐existing CVD, number of comorbidities, last observed BMI, last observed hypertension status and clustering within practices.

### 3.3 Effect modification of the association between remission and CVD outcomes

Table 3 presents the results of remission status‐subgroup variable interactions. There were no significant interaction effects for models on CVD events or macrovascular complications. The association between remission and microvascular complications was modified by age group and number of comorbidities. Stratifying by age group at baseline, remission was associated with lower risk of microvascular complications for all age groups, but this association was stronger for younger groups (eg aHR: 0.59 (0.41–0.84) and aHR: 0.78 (0.67–0.92) for those aged <45 years and 75–84 years, respectively). Stratifying by number of comorbidities, remission was associated with lower risk of microvascular complications for those with 0 or 1–2 comorbidities (aHRs: 0.65 (0.56–0.75) and 0.65 (0.58–0.73), respectively) compared to those with 3+ comorbidities (aHR:0.83 (0.69–0.99)). Associations for the different subgroups are presented in Table 4. Gender, BMI, pre‐existing CVD, duration of diabetes and HbA_1c_ level at baseline did not modify the association between remission and microvascular complications. Similar results were observed amongst those who did not have pre‐existing CVD, microvascular or macrovascular complications at baseline.

**TABLE 3:** Size and statistical significance of remission‐subgroup interactions on CVD events, microvascular complications, macrovascular complications^a^

Subgroup	*N*	CVD event		Microvascular complications		Macrovascular complications	
		Interaction HR (95%CI)	*p*‐Value	Interaction HR (95%CI)	*p*‐Value	Interaction HR (95%CI)	*p*‐Value
Age							
<45	3736	1.05 (0.90–1.21)	.545	1.09 (1.02–1.18)	.018	1.03 (0.92–1.15)	.624
45–54	8683						
55–64	14,381						
65–74	18,987						
75–84	13,696						
Sex							
Female	27,979	0.94 (0.66–1.33)	.712	0.93 (0.81–1.06)	.279	0.99 (0.74–1.32)	.934
Male	35,442						
Diabetes duration							
<5	23,915	0.91 (0.78–1.08)	.282	1.08 (0.97–1.19)	.158	0.95 (0.84–1.09)	.482
5 to 10	19,204						
10 to 20	16,501						
20+	3801						
Pre‐existing CVD							
No	55,829	0.96 (0.52–1.80)	.907	1.10 (0.87–1.41)	.424	1.11 (0.71 – 1.71)	.645
Yes	7592						
Total number comorbidities							
0	18,240	1.09 (0.86–1.37)	.468	1.16 (1.02–1.31)	.021	1.10 (0.91–1.33)	.313
1 to 2	35,189						
3+	9992						
BMI							
Normal/underweight (<25 kg/m^2^)	2348	0.99 (0.81–1.21)	.898	0.92 (0.83 −1.02)	.104	0.99 (0.84–1.18)	.929
Overweight (25–29.9 kg/m^2^)	6056						
Obese (≥30 kg/m^2^)	10,171						
HbA_1c_							
<6.0%	13,258	0.99 (0.87–1.11)	.823	1.00 (0.90–1.12)	.936	0.96 (0.86–1.07)	.473
6.0–6.4%	4682						
≥6.5%	45,481						

^a^ Cox regression models included the specified interaction term and additionally adjusted for age group, sex, diabetes duration, pre‐existing CVD, total number of comorbidities, last observed BMI, last observed hypertension status, baseline BMI and clustering within practices.

**TABLE 4:** Association between remission and incidence of microvascular complications over five‐year follow‐up in the CHIA type 2 diabetes cohort by subgroups^a^

	*N*	Microvascular complications	
		HR (95% CI)	*p*‐Value
Age <45	3736		
No remission	1		
Remission		0.59 (0.41–0.84)	.004
Age 45–54	8683		
No remission	1		
Remission		0.57 (0.45–0.73)	<.001
Age 55–64	14,381		
No remission	1		
Remission		0.66 (0.57–0.76)	<.001
Age 65–74	18,986		
No remission	1		
Remission		0.68 (0.59–0.78)	<.001
Age 75–84	13,696		
No remission	1		
Remission		0.78 (0.67–0.92)	.003
No comorbidities	17,031		
No remission	1		
Remission		0.65 (0.56–0.75)	.001
1–2 comorbidities	33,430		
No remission	1		
Remission		0.65 (0.58–0.73)	<.001
3+ comorbidities	9021		
No remission	1		
Remission		0.83 (0.69–0.99)	.035

^a^ Cox regression models included the specified interaction term and additionally adjusted for age group, sex, diabetes duration, pre‐existing CVD, total number of comorbidities, last observed BMI, last observed hypertension status, baseline BMI and clustering within practices.

## 4 DISCUSSION

In this large population‐based cohort of 60,287 people with type 2 diabetes, we found that remission was associated with a substantially lower risk of CVD outcomes including events, microvascular and macrovascular complications. The effect on microvascular complications was modified by age and number of comorbidities. Younger age and fewer comorbidities amongst those achieving remission were associated with a lower risk of microvascular complications. Sex, diabetes duration, pre‐existing CVD, BMI and HbA_1c_ level did not modify the remission‐CVD association.

This study extends the literature by examining the association between remission (in the absence of medication or surgery) of type 2 diabetes and CVD outcomes. Although the literature around glucose control and CVD outcomes (especially microvascular and macrovascular complications) is mixed,^15^, ^16^, ^17^ our findings are consistent with a few meta‐analyses from clinical trials examining the effect of intensive glucose control on CVD that reported glucose control was associated with a lower risk of CVD.^18^, ^19^ Remission is defined biochemically by lower glucose levels so it is plausible that these findings would be comparable.

In contrast with some previous studies, we do not find evidence of effect modification by pre‐existing CVD.^19^, ^20^, ^21^ Other studies have also reported no modification effect of pre‐existing CVD on the association between glucose control and CVD events.^22^ Although biochemical remission is comparable to tighter glucose control, differences in results could be explained by hypotheses in the literature, suggesting that the state of remission for at least a few months goes beyond biochemical glucose control to include reduced inflammation, decreased insulin resistance and enhanced gut hormone release, which may contribute to lower CVD risk.^23^, ^24^ It is thought that remission is linked to a reduction in adipokines such as leptin and inflammatory cytokines such as TNF‐α and several interleukins, as well as an increase in adiponectin concentrations, which reduces several cardio‐metabolic risk factors.^25^ Other explanations for these differences in the findings may be due to variations in study population such as greater diversity in age and comorbidities in the present study. Our findings on the effect of baseline BMI levels are in line with previous study that reported BMI level did not modify the association between HbA_1c_ and cardiovascular events in a Dutch population.^26^ However, this previous study looked at a much smaller population, of whom many had vascular disease at baseline, which may have explained the absence of significant associations.

Strengths of the study include the use of a large observational cohort with extended follow‐up, which allowed interaction effects to be examined, as well as the availability of data on comorbidities, medication and all HbA_1c_ measurements over the seven‐year duration of the study period. Limitations include the presence of missing data on variables which may reflect under‐testing or under‐reporting in routine data. Although we were able to use multiple imputation techniques which provide valid statistical inferences under the missing at random assumption, it is not possible to test for this assumption. Our data on medication were limited to specific periods of time (6‐month blocks), which may have misclassified some individuals’ medication exposure and therefore remission status. However, given the large size of the cohort, it is likely that any effect would be small. Exact date of death was also not available in the dataset which may have resulted in under or overestimation in time to event analyses. As with most studies using routine data, we were not able to distinguish between intentional or unintentional remission and did not have data on cause of death. It is possible that some of our findings may be confounded by unintentional remission, though some studies have found this to have minimal impact on outcomes.^27^, ^28^ Finally, our study population comprised mostly people of white ethnicity and findings may not be generalisable to more ethnically diverse populations.

Given the limited NHS resources and growing prevalence of type 2 diabetes, prioritising subgroups for targeted and personalised interventions is challenging. Our findings suggest that a focus on younger age groups and those with fewer comorbidities could be one approach with potential to reduce the impact of microvascular complications, as they are more likely to benefit most. Targeted interventions that focus on promoting remission as an achievable outcome in these groups could be justified given the potential to reduce the burden of microvascular complications and associated costs.

## CONFLICT OF INTERESTS

None to declare.

## AUTHOR CONTRIBUTION

**Hilda Hounkpatin:** Conceptualization‐Equal, Formal analysis‐Lead, Investigation‐Lead, Methodology‐Lead, Writing‐original draft‐Lead, Writing‐review & editing‐Equal. **Beth Stuart:** Funding acquisition‐Equal, Methodology‐Supporting, Writing‐review & editing‐Equal. **Andrew Farmer:** Conceptualization‐Equal, Funding acquisition‐Equal, Writing‐review & editing‐Equal. **Hajira Dambha‐Miller:** Conceptualization‐Lead, Funding acquisition‐Lead, Methodology‐Equal, Writing‐review & editing‐Equal.

## ETHICAL APPROVAL

CHIA is an anonymous National Health Service database, and all individuals have consented for collection of their medical records for inclusion in the database. Ethical and governance approval for this study was obtained from the University of Southampton (ERGO 56127), and Care and Health Information Exchange Information Governance Group (CHIE IGG).

## Data Availability

We do not have governance permissions to share individual‐level data on which these analyses were conducted since they derive from clinical record data. However, direct data requests can be made to the database (CHIA).
